# The role of mothers-in-law in antenatal care decision-making in Nepal: a qualitative study

**DOI:** 10.1186/1471-2393-10-34

**Published:** 2010-07-01

**Authors:** Bibha Simkhada, Maureen A Porter, Edwin R van Teijlingen

**Affiliations:** 1 School of Medicine & Dentistry, Division of Applied Health Sciences, University of Aberdeen, Aberdeen, Scotland, UK; 2 Centre for Midwifery, Maternal & Perinatal Health, University of Bournemouth, Bournemouth, UK

## Abstract

**Background:**

Antenatal care (ANC) has been recognised as a way to improve health outcomes for pregnant women and their babies. However, only 29% of pregnant women receive the recommended four antenatal visits in Nepal but reasons for such low utilisation are poorly understood. As in many countries of South Asia, mothers-in-law play a crucial role in the decisions around accessing health care facilities and providers. This paper aims to explore the mother-in-law's role in (a) her daughter-in-law's ANC uptake; and (b) the decision-making process about using ANC services in Nepal.

**Methods:**

In-depth interviews were conducted with 30 purposively selected antenatal or postnatal mothers (half users, half non-users of ANC), 10 husbands and 10 mothers-in-law in two different (urban and rural) communities.

**Results:**

Our findings suggest that mothers-in-law sometime have a positive influence, for example when encouraging women to seek ANC, but more often it is negative. Like many rural women of their generation, all mothers-in-law in this study were illiterate and most had not used ANC themselves. The main factors leading mothers-in-law not to support/encourage ANC check ups were expectations regarding pregnant women fulfilling their household duties, perceptions that ANC was not beneficial based largely on their own past experiences, the scarcity of resources under their control and power relations between mothers-in-law and daughters-in-law. Individual knowledge and social class of the mothers-in-law of users and non-users differed significantly, which is likely to have had an effect on their perceptions of the benefits of ANC.

**Conclusion:**

Mothers-in-law have a strong influence on the uptake of ANC in Nepal. Understanding their role is important if we are to design and target effective community-based health promotion interventions. Health promotion and educational interventions to improve the use of ANC should target women, husbands and family members, particularly mothers-in-law where they control access to family resources.

## Background

Improving maternal health is the fifth of eight Millennium Development Goals (MDGs), aiming to reduce the maternal mortality ratio (MMR) by three quarters between 1990 and 2015 [1]. There are an estimated 281,500 maternal deaths worldwide each year, over 99% of which occur in the developing world and most are avoidable [2]. In Nepal, maternal mortality is estimated at 281 per 100,000 live births, which is one of the higher MMRs in the world. Underutilisation of health services is one factor contributing to high maternal mortality, e.g. 81% of births take place at home, many without skilled attendants [3]. Complications during pregnancy and childbirth such as haemorrhage, sepsis, spontaneous abortion, pre-eclampsia and eclampsia, and prolonged/obstructed labour are the leading causes of death and disability among women of reproductive age in developing countries [4]. Millions of women lack access to adequate care during pregnancy.

Antenatal care (ANC) is an important determinant of safe delivery that can offer opportunities to encourage women to deliver with a skilled attendant in a health facility [5] ultimately helping to reduce maternal mortality [6]. A key function of ANC is to offer health information and services that can significantly improve the health of women and their infants [7]; moreover ANC usage has a positive impact on the uptake of postnatal services [8]. Recently, the potential of the antenatal period for the prevention of HIV transmission from mother to child has led to renewed interest in access to and use of antenatal services [9]. The World Health Organization (WHO) recommends at least four ANC visits since empirical evidence has shown that this is sufficient for uncomplicated pregnancies [10].

A recent systematic review has identified factors affecting the uptake of ANC services in developing countries [11]. Maternal education [12], husband's education, marital status, service availability [13], cost [14], household income, women's employment, media exposure [15] and having a history of obstetric complications affected ANC uptake in most developing countries. Cultural beliefs and ideas about pregnancy also had an influence on ANC use in some countries. Higher parity had a negative effect on adequate attendance [14]. A number of studies found relationships between economic factors and ANC utilisation, and identified cost as a barrier to uptake [11].

### ANC in Nepal

The Government of Nepal aims to increase the percentage of pregnant women attending four antenatal visits to 80% [16]. The uptake of ANC in Nepal is low, with only 29% of pregnant women receiving the recommended four check-ups and 26% not receiving any [3]. This figure is not only well below the government target, it is also below the developing countries' average of 68%. The average for the region is 54% in South Asia and 82% in East Asia [7]. With limited resources, inadequate administrative capacity and competing health needs, the Government of Nepal struggles to provide even minimal ANC [17].

### Mother-in-law's role

A few studies have examined socio-demographic and socio-economic factors in the uptake of ANC in Nepal [12,18], finding that better educated or economically advantaged women are more likely to receive ANC. Evidence from similar contexts such as Pakistan and Bangladesh suggests that mothers-in-law [19,20], and other family members significantly affect uptake [21]. Older women, especially mothers-in-law did not consider ANC essential during pregnancy and often discouraged their daughters-in-law from attending in rural Bangladesh [20]. Women who perceived friends and family to be supportive were twice as likely to attend ANC in Jamaica [22]. In Nepalese society it is customary for senior women to occupy the top position in a hierarchical family network, exercising authority and power over daughters-in-law. In this patriarchal society, decisions about the management of pregnancy and childbirth usually come within the purview of older women, especially mothers-in-law [23] as do those about perinatal care [24,25]. Therefore, our study set out to examine in detail the role of mothers-in-law in decision-making and uptake of ANC by their daughters-in-law, gaining insight into the circumstances that affect the decision-making process around ANC.

## Methods

The dominant method in this mixed-methods study is qualitative. Qualitative research is commonly used in studies of health service organisation [26]; for example, it has been used to explore women's use of ANC [27] and gain understanding of how mothers-in-law view their role within child birthing [24]. Qualitative semi-structured interviews offer opportunities for collecting unexpected valuable data [28]. Given the exploratory nature of this research amongst an undeveloped and understudied population which included mothers-in-law and husbands, in-depth interviewing helped to draw out contextual, descriptive and process-oriented information and allowed respondents to identify issues from their own perspectives, providing rich and detailed information about their perceptions and experiences [29]. In addition a small questionnaire was used to collect background information and demographic data from the interviewees. The intention was to compare the accounts of users and non-users of ANC and of women and other family members (mothers-in-law and husbands). Equal numbers of users (women who had at least one antenatal visit) and non-users (women who had no ANC in the index pregnancy) were purposively selected by locality (rural/urban), education, age, ethnicity and religion. Women in early pregnancy (before six months) were excluded as their ANC use was incomplete.

Local health workers initially helped to identify women, but due to the lack of systematic record-keeping on pregnant and postnatal women we used a social network approach in the community to recruit interviewees. As pregnant women and new mothers were the primary focus of the study, recruitment stopped when data saturation had been reached [30]. Altogether thirty in-depth interviews were conducted with pregnant women or women who had a child younger than one year. Due to constraints of time and money we decided beforehand to interview ten husbands and ten mothers-in-law, to include family members of ANC users and non-users. Fieldwork was conducted in semi-urban and rural areas of Nepal, each about 20 km from the capital Kathmandu. Data collection began at the end of 2006 and continued for six months. Most of the interviews occurred in participants' homes, but some were in school halls or health clinics.

A short semi-structured questionnaire and interview schedule were developed in Nepali. Both were piloted on a pregnant woman, a mother of a child under the age of one, a mother-in-law and a husband [31]. The questionnaire was used to ascertain socio-demographic information about respondents (copy available from first author), reducing the length of the in-depth interview and guiding its content. As we planned a predominantly qualitative study with relatively low numbers of interviewees it was envisaged that no statistical analysis would be conducted and the construct validity of the instrument was a lesser concern. The questions were largely demographic and derived from the DHS (Demographic and Health Surveys) questionnaire. As most of the Nepalese women were illiterate, the interviewer completed the questionnaire with them.

Interviews were conducted by the first author who is native Nepali and audio-recorded with respondents' permission. Recordings were transcribed and translated into English by the first author single-handedly as suggested by Twinn [32]. Two English transcripts were translated back [33] into Nepali at a later date to check the quality of translation. Data analysis was concurrent with data collection to identify when data saturation was reached. Inductive thematic analysis [34] is a coding process which involves systematically sorting textual data into categories from which patterns emerge and themes develop. With 50 interviews computer-assisted analysis becomes more viable [35], hence transcripts were coded using N-Vivo software [36]. To ensure the trustworthiness of coding and interpretation, ten transcripts were randomly selected and coded independently by MP and EvT and any divergences discussed. Verbatim quotations are used to illustrate the range of perceptions within each theme from the respondent's perspective.

Ethical approval (Ref no-1052) was obtained from the Nepal Health Research Council (the national ethical clearance body). Participants were fully informed about the nature of the study and assured of anonymity. Due to high levels of illiteracy, verbal consent was taken from all respondents prior to the interview.

## Results

Socio-demographic characteristics of the 50 respondents are presented in Table 1. This shows some major social differences between users and non-users. The majority of the non-users were older women with higher parity and mainly from lower class/caste. They were also more likely to have illiterate husbands and to be illiterate themselves. All of the mothers-in-law were illiterate and most were over 60.

**Table 1:** Socio-demographic characteristics of respondents

Socio-demographic characteristics of women (N = 30)			
**Variable**	**Categories**	**ANC user (N = 15)**	**ANC non-user (N = 15)**

**Age**	Below 20	3	5
	
	21-25	11	2
	
	26 and over	1	8

**Number of children**	None but pregnant	8	4
	
	2 or fewer	4	2
	
	3 or more	2	6

**People in family home**	below 5	5	2
	
	6 to 12	10	13

**Women's education**	Literate	11	2
	
	Illiterate	4	13

**Socio-demographic characteristics of husbands (N = 10)**			

**Education**	Literate	5	1
	
	Illiterate	0	4

**Age**	<30 years	4	2
	
	>30 years	1	3

**Socio-demographic characteristics of mothers-in-law (N = 10)**			

**Education**	Literate	0	0
	
	Illiterate	6	4

**Age**	< 60 years	2	0
	
	> 60 years	4	4

Five important sub-themes emerged from the analysis: workload of pregnant women, mothers-in-law's perceptions, past experiences, control over resources, and family relationships and power.

### Pregnant women's workload

Most of the women said that they could not attend ANC check-ups due to their workload in the home, which they perceived as heavy and unavoidable. According to some non-users, their mothers-in-law prioritised household work over their daughters-in-law's health. Some of the non-users had used ANC in past pregnancies; one such non-user said that her mother-in-law viewed ANC merely as a diversion and described her experience during her only antenatal visit:

"My in-laws and my husband scolded me. I thought it would be better not to go for check-ups than to hear criticism from them. I went for a check-up once in my previous pregnancy; I had to wait in the queue. They blamed me for going to see my friends for entertainment, leaving my household work undone" (Non-user Woman1).

Many women said that their mothers-in-law's expectation that household work had to be done made it hard for them to go for ANC check-ups without finishing tasks assigned to them.

"My mother-in-law doesn't help. It might be due to her past experiences. She used to do all the work by herself during her time of pregnancy. So she wants me to do the same. I have lots of work here at home, so I don't go for ANC check-ups" (Non-user Woman 4).

However, heavy workloads were also an issue for some women who had managed to use ANC. The key factors appear to be a combination of their husbands' literacy and having a source of income; living close to a health institution which offered ANC also helped. However, a user woman who had a heavy burden of household work said that she was not treated well by her mother-in-law when she could not finish her work as a result of an ANC visit.

"I had to do lots of household work. I had to go to the forest to collect wood and grass for cattle and also do housework. I went to the forest even after nine months of pregnancy. My mother-in-law scolded me when I left household work behind due to the health post visit... it would be better if pregnant women didn't have to work so much and could get rest when they wished" (User Woman 10).

The majority of the ANC user husbands accepted that women have to work hard during pregnancy in Nepalese society. Several expressed the view that mothers-in-law usually played an unsupportive role, providing much work which had to be completed before they considered ANC attendance. For example;

"In the village women work until delivery; they can't stay without working. It's not like in the city..... I have seen some of the mothers-in-law scold them, saying that they hadn't taken a rest in their own time and that the daughter-in-law doesn't need to rest. Some mothers-in-law do not tell their daughters-in-law to go for antenatal check-ups. Women have to finish their work before they go" (User Husband 1).

Most mothers-in-law expected their daughters-in-law to work during pregnancy as they had worked themselves. As they perceived the workload for their daughters-in-law to be lighter than in their own time, they had little sympathy for them, believing the earlier tradition to be better. A user mother-in-law said that working hard is part of the culture for pregnant women, implying that it was beneficial.

"In the past, pregnant women had to work very hard. We never got time to take rest. We had to be strong and carry our baby during work. But pregnant women nowadays take rests most of the time. They do not work hard and they do not carry their baby during work" (ANC users' Mother-in-law 2).

There is a cultural practice in extended families of sending pregnant women to their maternal home, especially when they are unwell. Women mentioned that daughters were sometimes looked after and treated better in their maternal home compared to the care and food which they received in their in-laws' house. Women felt that they should be able go to their maternal home if they wished to take a rest during pregnancy as less demands were made of them there.

"I get a lot of rest in my mother's home. My sister-in-law and mother do most of the work. Better food as well... people in my maternal home understand my problem better than the in-laws do" (Non-user Women 4).

Several women mentioned mothers-in-law's belief that hard work is acceptable as pregnancy is not an illness.

### Mother-in-law's perceptions and their own experiences of ANC

A theme emerging from both husbands' and women's accounts concerned mothers-in-law's perceptions of the benefits of ANC and their own experience of pregnancy and delivery. ANC was viewed as a curative rather than preventative form of care. Most mothers-in-law believed that as pregnancy is a natural state, there was no need to seek medical care and that ANC was only necessary when complications occurred. Some could see no benefits in ANC, especially if they had not experienced problems during their own pregnancies or deliveries. Often they relied on traditional medicine for any illness and there was no common history of attending ANC.

A non-user woman related how her mother-in-law decried the benefits of ANC when she mentioned the need for a nutritious diet during pregnancy:

"My mother-in-law said that pregnant women didn't go for antenatal check-ups in the old days. She told me that she had all her children without any antenatal check-ups and all are fine. She questioned why different foods and antenatal check-ups are necessary for pregnant women. That's why I didn't go" (Non-user Woman 1).

Mothers-in-law's own experiences of pregnancy influenced their daughters-in-law's uptake of ANC. Women believed that mothers-in-law did not want to send their daughters-in-law because they had all their children without any antenatal or medical care. Their explanations showed that they sometimes internalised these views:

"A health volunteer told me that I had to go for antenatal check-ups ... I heard that women hadn't gone for antenatal check-ups in the old days but had their baby without check-ups. My mother-in-law told me the same" (Non-user Woman 4).

Similarly, a non-user's husband appeared to dismiss ANC on the basis of his mother's views of its usefulness and that of other mothers-in-law known to him.

"Some mothers-in-law say nothing will happen without any antenatal check-ups and that they gave birth to babies without going to hospitals" (Non-user Husband 3).

Mothers-in-law in families of higher status in the community sometimes experienced pressure to send their daughters-in-law for ANC although they personally believed it to be unnecessary. Whereas few mothers-in-law got pressure from their sons to send their wives for ANC, several mothers-in-law felt pressure from health workers (Female Community Health Volunteer) living in their community. For example:

"I don't know anything. I never went for check-ups during my pregnancies. If everybody is saying pregnant women should go for check-ups then my daughter in-law should go as well" (ANC user's Mother-in-law 1).

A few mothers-in-law talked negatively about the old days when money and attitudes prevented people from seeking ANC. One mother-in-law wanted to improve her grandchildren's chances and encouraged her daughter-in-law to attend. She had only one son and was concerned about future generations.

"I told my son to take my daughter-in-law to the hospital for check-ups ...It was very different in my time. I never went for antenatal check-ups. It was a matter of shame and also we couldn't get enough money to go to hospital for check-ups" (ANC user's Mother-in-law 5).

One user's mother-in-law questioned the mother-in-law's role and influence, especially in the extended family where pregnant women get so much work. She believed that most mothers-in-law are barriers in the uptake of ANC for their pregnant daughters-in-law and supported hers by providing less work than she had personally experienced.

"I think the mother-in-law is the key person for daughters-in-law .... I think mothers-in-law should understand first, then the husband. It will be easier for women if the mother-in-law understands her problems" (ANC user's Mother-in-law 6).

A user woman said that her supportive mother-in-law helped her to go for check-ups but did not encourage hospital delivery because of the cost involved.

"My mother-in-law recommended attending antenatal check-ups. She already knew about them. She hadn't had check-ups in her time but advised me to go in case anything happens. I used to ask my mother-in-law and she gave me suggestions" (User Woman 7).

### Mother-in-law's power and control over resources

Power and control over resources are interrelated. Traditionally Nepalese mothers-in-law are powerful as they can access household resources through their close contact with the main family earners (their husbands and sons). Some women highlighted that their mothers-in-law's control over resources prevented them from receiving ANC. They also believed that mothers-in-law see no immediate benefits from ANC and considered the cost involved in preventive care as unnecessary. Lack of access to resources is a major barrier, especially where mothers-in-law control household finances:

"My personal expenses were under the control of my mother-in-law and even my husband had to ask her for money" (Non-user Woman 3).

Women's low status within households prevents them from keeping any income they might earn themselves. Most rural women did not have earnings of their own and this prevented them from accessing ANC. A non-user woman said that her lack of control over household resources resulted in her being unable to hire people to finish the work she could not do alone.

"It was due to lack of money and my work. There was a problem at home and I did not have money either. I asked my family but they said that it was time for work" (Non-user Woman 6).

A user woman also reported that mothers-in-law can make access to ANC difficult if they are concerned about costs. She mentioned that her mother-in-law favoured ANC as a result of pressure from health workers. Though she lived next to the private community hospital, she was sent to a distant health post because it was cheaper.

"My mother-in-law told me that they didn't have a tradition of going for antenatal check-ups. Now time is changing and you have the opportunity ... My mother-in-law used to cook dinner if I was not feeling well. She made an early meal for me if I had to go to the jungle for firewood. She cared for me well most of my pregnancy but she let me down in the last stage. She used to tell me that she did not have money because my husband never earned money" (User Woman 9).

Where they were the main income holder, mothers-in-law controlled their savings from the household income and often made daughters-in-law ask for money to go for ANC checks. A few mothers-in-law were positive and helped their daughters-in-law attend ANC check-ups.

"My mother-in law suggested I go for antenatal check-ups. She has some savings in her bank account and she gives me money for check-ups as well. I shared every problem with her" (User Woman 1).

Women have little or no power in their marital home and are almost entirely at the mercy of their mothers-in-law's perception of their pregnancy and delivery care needs. Most women and their husbands believed that mothers-in-law liked a commanding role as a result of being treated badly by their own mothers-in-law in earlier times. A user husband spoke about the dependent relationship of many daughters-in-law.

"Pregnant women ....do have problems at home. Generally, the in-laws don't tell their daughters-in-law to go for antenatal check-ups. They can't go without the permission of their in-laws" (User Husband 1).

Mothers-in-law appeared to have less influence on ANC uptake if they did not live in the same household as their daughters-in-law, the woman herself and her husband appearing to make the decision then.

"I didn't go for ANC check-ups in my time. Some of my friends told me that they should be done. I think it would be better if pregnant women go for ANC check-ups. I was always supportive of ANC but my daughter-in-law didn't want to go" (ANC non-users' Mother-in-law 1).

One mother-in-law who had health-related training (Traditional Birth Attendant and Health Volunteer) wanted to send her daughter-in-law for ANC but met resistance:

"The Health Assistant has asked me to bring my daughter-in-law for check-ups, but she did not listen to me. I could not force her .... We brought iron tablets for her but she did not take them" (ANC non-user's Mother-in-law 2).

It appeared in this case that the mother-in-law had less influence over her daughter-in-law than other mothers-in-law because they were not living in the same household.

### Mother and daughter-in-law's relationship

Poor communication and relationships between mothers-in-law and daughters-in-law were highlighted by some mothers-in-law, husbands and women as influencing the use of ANC. One non-user's mother-in-law who was positive about ANC, accompanied her own daughter but not her daughter-in-law as she considered that their relationship was poor.

"If she likes to go, she goes even if I tell her not to go. She never asked for any money from us. I think she asked someone outside. I do not even know if her children have been vaccinated. She never asks before she leaves home.......... She never speaks with me and I do not know a thing about her" (ANC non-user's Mother-in-law 4).

Another mother-in-law showed negative attitudes towards her daughter-in-law and was dissatisfied with her. She was against the use of ANC and was even unhappy that her son was visibly supporting his wife.

"My son is always behind his wife. He is not only helping, but also supporting her all the time" (ANC non-user's Mother-in-law 3).

Another non-user said that her mother-in-law was unsympathetic and would not fund ANC whatever her health condition might be.

"She does not like me to go for check-ups, even if I am unwell" (Non-user Woman 8).

A user woman, who had lost her baby in childbirth, had received some relief from housework during pregnancy, but did not get support to go to hospital for the delivery. She acknowledged that poor relationships with mothers-in-law could be a barrier to ANC.

"Some women might not have good relations with their mothers-in-law and so cannot manage to go for ANC" (User Woman 9).

A supportive mother-in-law also stated that a good relationship with daughters-in-law is essential during pregnancy. She believed that most mothers-in-law created problems for their daughters-in-law, but some daughters-in-law did not try to understand the mother-in-law's position in order to build a good relationship.

"Pregnant women should get love and affection from the family. In most cases, mothers-in-law don't like daughters-in-law. I think that won't be good for either of them" (ANC users' Mother-in-law 3).

Furthermore a user husband who had observed the relationship between his mother and his wife believed that family members should support pregnant women.

"My mother behaved well but sometimes the relationship between my mother and wife was not good. It's normal to have both good and bad aspects living in the same family but if mothers-in-law don't behave well with daughters-in-law at home, then there will be problems" (User Husband 1).

The above quotes clearly illustrate the difficulties that can arise within families where resources are in short supply and expectations cannot always be met. ANC is one of many competing interests and can become a battleground.

## Discussion

This qualitative study shows that mothers-in-law are perceived as having an influential role in the uptake of ANC in Nepal. As an important member of the family hierarchy, her active role in decision-making went largely unchallenged. In most cases, mothers-in-law seemed not to be in favour of ANC and played a negative role. However a few, usually more knowledgeable mothers-in-law, were positive and supported ANC check-ups. By their own accounts and also those of daughters-in-law and sons, mothers-in-law's attitudes towards their daughters-in-law's ANC, were influenced by their own past experiences, the household work expected of daughters-in-law and their perceptions of the need for and benefits of ANC. Their control over resources and power over subordinate family members also affected decision making about ANC.

Most Nepalese women carry a heavy workload as they have multiple roles at home. Mothers-in-law, particularly in rural areas, still tend to prioritise housework rather than antenatal or preventive care. Most mothers-in-law think pregnancy is a natural process which does not require medical care. In rural families, household chores and childcare are physically challenging full-time tasks, taking into account the average family size and basic living conditions. Significantly, the socio demographic information suggests that non-users had more extended family and therefore more work to do at home. This restricts women's ability to take part in other social activities including health care. Other studies concordantly describe how women's daily activities such as water-fetching in South Africa [37] or working in the fields in rural China [38] interfere with health seeking. Women's status within households is low and in rural Nepal daughters-in-law are expected to work longer and harder than other people, even when pregnant [9].

Most mothers-in-law rightly assumed that ANC requires a lot of time waiting in a queue. Long waiting times were also a barrier to ANC in Zimbabwe [27]. As they perceive ANC care to be unnecessary and a waste of time, they may discourage daughters-in-law from attending. This lack of support could also be due to poor knowledge about the importance of preventive care as all mothers-in-law are uneducated [39]. Previous studies suggest that women's [40,41] and their husbands' education [13,15] are the best predictors of ANC uptake. Husband's education is a stronger predictor than the woman's in the Philippines [42].

As all mothers-in-law in our study were illiterate, as are nearly all women of their generation, and were unlikely to have had ANC themselves, it is interesting establish why despite this personal history some still supported their daughters-in-law in accessing ANC. Mothers-in-law who had health-related training (e.g. as Female Community Health Volunteers) were more supportive of ANC. Studies in other groups (for example Traditional Birth Attendants) suggest that health-related training has a positive impact on health service utilization [43]. However, some mothers-in-law are supportive of women's use of ANC because of available financial resources or knowledge of the benefits of ANC. Living in a separate household sometimes meant mothers-in-law having less influence, improving the relationship between daughters-in-law and mothers-in-law, and encouraging a positive attitude towards ANC.

Traditionally in Nepalese culture women reside with their husband's family after marriage, and during pregnancy they depend entirely upon their mothers-in-law [9,44] since male involvement in maternity care is not common practice in Nepalese culture [45]. Mothers-in-law are given more responsibility and authority to allocate resources to their daughters-in-law in the belief that they understand women's issues, including health problems. Mothers-in-law have strong decision-making powers during pregnancy and delivery as husbands generally know little about childbirth [46]. A gender and age hierarchy also dictates that the authority to make pregnancy-related decisions is located in the mother-in-law in Pakistan [19] and India [47]. Interestingly, it is different in nuclear families in Nepal where decisions are predominantly made by husbands. Hence the ability of young women to make decisions is limited in Nepal [25,48] and in South Asia [24]. Daughters-in-law have less freedom but more responsibility than the mistress of the household and are not treated differently because of pregnancy. It is believed that daughters-in-law should eat only after they have fed all other family members [49].

This study suggests that mothers-in-law's control over available resources in the household can prevent daughters-in-law obtaining ANC. Daughters-in-law who work but have no control over the use of their earnings also experience difficulty accessing ANC, though a recent study has highlighted that women who have control over resources are better able to make use of health care services [50]. Often decisions about health care expenses are controlled by other family members and limit women's access to health care in Nepal [51] and in other developing countries [52]. It would be easy to conclude, as other authors have done [39], that pregnant women in Nepal lack 'agency' i.e. "the ability to define one's goals and act upon them" [53]. Kabeer [[54]: p438] highlights that agency can also take the form of "bargaining and negotiation, deception and manipulation, subversion and resistance as well as ... reflection and analysis". Although many of our quotes confirm that mothers-in-law were the key decision-makers, we also found elements of 'resistance'. For example, a mother-in-law bemoaned the fact that her daughter-in-law would attend for ANC even if she advised against; another deduced that her daughter-in-law had obtained the necessary funds elsewhere. These instances suggest that even in a hostile environment, pregnant women can sometimes have a sense of agency which enables them to exert control over their health.

The relative poverty of the household may mean mothers-in-law do not see the immediate benefit of spending money on preventive care. Similarly poor relationships between daughters-in-law and mothers-in-law may influence whether money is spent on activities other than health care. Mumtaz and Salway [19] showed that the strength of individual women's relationships with other key family members, including mothers-in-law, were significant factors in determining whether or not resources were directed towards ANC.

Mothers-in-law's perceptions of the benefits of ANC emerged as an important factor in its uptake in our study. Most mothers-in-law believed ANC was essential only in the case of complications. According to Onega [55] perceptions involve individuals' beliefs about their susceptibility to disease, as well as the seriousness with which they view the threat of illness. These perceptions, attitudes, beliefs and values may contribute to mothers-in-law not encouraging their daughters-in-law to receive ANC. Evidence from countries like India, suggests that routine ANC is perceived as curative rather than preventive [56]. Similarly, mothers-in-law were reported not to believe that a nutritious diet was beneficial during pregnancy, which may be due to lack of knowledge/awareness or a mask for financial constraints.

### Strengths and limitations of the study

A strength of this study is that it examines the perceptions of a range of participants who have a role in ANC uptake, not only interviewing women but also their mothers-in-law and husbands and thereby recognising the cultural context of rural women in Nepal. The first author (BS) is a native speaker whose understanding of the local dialect and translation skills have provided rich data for analysis. However when Nepali is translated into English subtleties in the meaning of local dialogues are sometimes lost or difficult to convey. Although people's spoken language does not always follow conventional grammatical rules, we have tried to convey the original meaning, which occasionally leads to quotes appearing ungrammatical. Birbili suggests that the quality of translation in a research setting depends on (a) the autobiography of translators; (b) the linguistic competence of the translators, (c) the translators' knowledge of the people under study; and (d) the circumstances in which the translation takes place [57]. Since the translation in this study was conducted by the lead author, who is both a native Nepali speaker and the key interviewer, the circumstances surrounding the translation were considered optimal.

A limitation of this study is that although the women were interviewed in a rural area, this is by no means very remote in Nepal. Women could, and occasionally had travelled to Kathmandu for emergency health treatment. A further limitation of this study is that only 10 husbands and 10 mothers-in-law could be included owing to time and financial constraints. Purposive sampling resulted in significant differences in socio-demographic characteristics between users and non-users which influenced the perception of value and uptake of ANC services but could not be fully explored in a small sample. Convenience sampling meant that most mothers-in-law included were aged around 60 and all were illiterate. It was not feasible to include literate mothers-in-law as literacy is rare in that generation, especially among rural communities [58]. Thus we are unable to explore the relationship between literacy and uptake of ANC. As mothers-in-law were all illiterate and had no experience of ANC themselves, there is a clear generation gap which could not be easily separated in the analysis to assess their influence on ANC uptake.

This study suggests that most respondents recognised the influential role that mothers-in-law played in their daughters-in-law's uptake of ANC. This involvement could be challenging, as their perception of the need for ANC is shaped by their own past experiences of pregnancy without medical intervention, of which they are very proud [45]. Therefore health promotion activities and health interventions aimed at increasing awareness of the value of ANC need to be targeted at mothers-in-law as well as women themselves to facilitate behaviour change. This study suggest that engaging in a positive and innovative way with mothers-in-law and establishing relationships of respect, is one of the first steps needed if change is to occur [59], given the hierarchy of power and social roles in rural Nepal. Educating mothers-in-law in this way may also have the effect of increasing their awareness of the heavy burden of household work which many young women carry in rural communities.

## Conclusion

The study concludes that the use of ANC in Nepal is strongly influenced by mothers-in-law's roles and attitudes. Sometimes this influence was positive, encouraging women to seek ANC but more often it was negative. If ANC uptake is to improve, women's family members, especially their mothers-in-law must be engaged and educated about the benefits of ANC and appropriate care-seeking behaviour during pregnancy. At the same time health promoters in the field of ANC need to remember that there is a generation gap, as many women of the older generation are illiterate and did not experience any ANC themselves when they were pregnant. At the policy level this study suggests that mothers-in-law's involvement can have a positive effect on ANC utilisation, which may also improve other aspects of health care such as postnatal and neonatal care in Nepal. Further studies which examine women's experiences of health service providers and health institutions may improve understanding of the reasons for poor ANC utilisation.

## Competing interests

The authors declare that they have no competing interests.

## Authors' contributions

BS, EVT and MP were responsible for the study conception and design and BS was responsible for the drafting of the manuscript. BS performed the data collection and BS, EVT and MP performed the data analysis. BS provided administrative support. BS, EVT and MP made critical revisions to the paper. EVT and MP supervised the study. All authors read and approved the final manuscript.

## Pre-publication history

The pre-publication history for this paper can be accessed here:

http://www.biomedcentral.com/1471-2393/10/34/prepub

## References

[B1] UN The Millennium Development Goals Report 2008 2008 United Nations Department of Economic and Social Affairs. New York

[B2] Hogan MC Foreman KJ Naghavi M Ahn SY Wang M Makela SM Lopez AD Lozano R Murray CJL Maternal mortality for 181 countries, 1980-2008: a systematic analysis of progress towards Millennium Development Goal 5 The Lancet 2010 375 9726 1609 23 (Online). 10.1016/S0140-6736(10)60518-1 20382417

[B3] DHS Nepal demographic and Health Survey 2006 2007 Ministry of Health and Population Government of Nepal, New ERA Kathmandu Nepal and Macro International Inc. USA

[B4] WHO The World Health Report: make every mother and child count 2005 World Health Organization, Geneva

[B5] Mrisho M Obrist B Schellenberg JA Haws RA Mushi AK Mshinda H Tanner M Schellenberg D The use of antenatal and postnatal care: perspectives and experiences of women and health care providers in rural southern Tanzania BMC Pregnancy and Childbirth 2009 9 10 19261181 10.1186/1471-2393-9-10 PMC2664785

[B6] Nuraini E Parker E Improving knowledge of antenatal care (ANC) among pregnant women: a field trial in central Java, Indonesia Asia Pacific Journal of Public Health 2005 17 1 3 8 10.1177/101053950501700102 16044824

[B7] WHO, UNICEF Antenatal Care in Developing Countries: Promises, Achievements and Missed Opportunities: An Analysis of Trends, Levels, and Differentials: 1990-2001 2003 Geneva and New York: WHO & UNICEF

[B8] Chakraborty N Islam MA Chowdhury RI Bari W Utilisation of postnatal care in Bangladesh: evidence from a longitudinal study Health & Social Care in the Community 2002 10 6 492 502 10.1046/j.1365-2524.2002.00389.x 12485137

[B9] UNICEF Situation of children and women in Nepal 2006 http://www.un.org.np/reports/UNICEF/2006/situation-analysis/conver-page.pdf The United Nations Children's Fund. Accessed on 20/02/08.

[B10] Villar J Ba'aqeel H Piaggio G Lumbiganon P Miguel BJ Farnot U Al-Mazrou Y Carroli G Pinol A Donner A Langer A Nigenda G Mugford M Fox-Rushby J Hutton G Bergsjø P Bakketeig L Berendes H Garcia J for the WHO Antenatal Care Trial Research Group WHO antenatal care randomised trial for the evaluation of a new model of routine antenatal care The Lancet 2001 357 9268 1551 1564 10.1016/S0140-6736(00)04722-X 11377642

[B11] Simkhada BD van Teijlingen ER Porter M Simkhada P Factors affecting the utilisation of antenatal care in developing countries: systematic review of the literature Journal of Advanced Nursing 2008 61 3 244 60 18197860 10.1111/j.1365-2648.2007.04532.x

[B12] Matsumura M Gubhaju B Women's status, household structure and the utilisation of maternal health services in Nepal Asia-Pacific Population Journal 2001 16 1 23 44

[B13] Nielsen BB Hedegaard M Liljestrand J Thilsted SH Joseph A Characteristics of antenatal care attenders in a rural population in Tamil Nadu, South India: A community-based cross-sectional study Health & Social Care in the Community 2001 9 6 327 333 10.1046/j.1365-2524.2001.00310.x 11846810

[B14] Overbosch G Nsowah-Nuamah N van den Boom G Damnyag L Determinants of antenatal care use in Ghana Journal of African Economies 2004 13 2 277 301

[B15] Pallikadavath S Foss M Stones RW Antenatal Care: Provision and Inequality in Rural North India Social Science & Medicine 2004 59 6 1147 1158 10.1016/j.socscimed.2003.11.045 15210087

[B16] MOHP Second Long term Health Plan 1997-2017 2009 Ministry of Health and Population, Kathmandu http://mohp.gov.np/english/publication/second_long_term_health_plan_1997_2017.php Accessed on 06-11-2009.

[B17] Simkhada BD van Teijlingen ER Porter M Simkhada P Major problems and key issues in Maternal Health in Nepal Kathmandu University Medical Journal 2006 4 2 258 263 18603913

[B18] Sharma B Utilisation of Antenatal Care Services in Nepal Nepal Population Journal 2004 11 10 79 97

[B19] Mumtaz Z Salway SM Gender, pregnancy and the uptake of antenatal care services in Pakistan Sociology of Health & Illness 2007 29 1 1 26 17286703 10.1111/j.1467-9566.2007.00519.x

[B20] Chowdhury AMR Mahbub A Chowdhury AS Skilled attendance at delivery in Bangladesh: an ethnographic study Research Monograph Series 2003 Research and Evaluation Division, BRAC Dhaka, Bangladesh 22

[B21] Erci B Barriers to utilization of prenatal care services in Turkey Journal of Nursing Scholarship 2003 35 3 269 273 10.1111/j.1547-5069.2003.00269.x 14562496

[B22] McCaw-Binns A La Grenade J Ashley D Under-users of antenatal care: a comparison of non-attenders and late attenders for antenatal care, with early attenders Social Science & Medicine 1995 40 7 1003 1012 10.1016/0277-9536(94)00175-S 7792624

[B23] Mullany BC Hinde MJ Becker S Can women's autonomy impede male involvement in pregnancy health in Kathmandu, Nepal? Social Science & Medicine 2005 61 9 1993 2006 10.1016/j.socscimed.2005.04.006 15922498

[B24] Masvie H The role of Tamang mothers-in-law in promoting breast feeding in Makwanpur District, Nepal Midwifery 2006 22 1 23 31 10.1016/j.midw.2005.02.003 15967547

[B25] Mesko N Osrin D Tamang S Shrestha BP Manandhar DS Manandhar M Standing H Costello AM Care for perinatal illness in rural Nepal: a descriptive study with cross-sectional and qualitative components BMC International Health and Human Rights 2003 3 3 10.1186/1472-698X-3-3 12932300 PMC194728

[B26] Pope C Mays N Pope C, Mays N Qualitative methods in health research 2006 Third Blackwell Publishing 1 11 Qualitative research in health care.

[B27] Mathole T Lindmark G Majoko F Ahlberg BM A qualitative study of women's perspectives of antenatal care in a rural area of Zimbabwe Midwifery 2004 20 2 122 132 10.1016/j.midw.2003.10.003 15177855

[B28] Coffey A Atkinson P Making Sense of Qualitative Data: Complementary Research Strategies 1996 Sage, London

[B29] DiCicco-Bloom B Crabtree BF The qualitative research interview Medical Education 2006 40 4 314 321 10.1111/j.1365-2929.2006.02418.x 16573666

[B30] Glaser BG Strauss AL The Discovery of Grounded Theory: Strategies for Qualitative Research 1967 Aldine Publishing Company, Chicago

[B31] van Teijlingen ER Hundley V The importance of pilot studies Social Research Update 2001 35 http://sru.soc.surrey.ac.uk/SRU35.html Accessed on 15/5/2010.

[B32] Twinn S An exploratory study examining the influence of translation on the validity and reliability of qualitative data in nursing research Journal of Advanced Nursing 1997 26 2 418 423 10.1046/j.1365-2648.1997.1997026418.x 9292378

[B33] Small R Yelland J Lumley J Rice PL Cotronei V Warren R Cross-cultural research: trying to do it better. 2. Enhancing data quality Australian and New Zealand Journal of Public Health 1999 23 4 390 395 10.1111/j.1467-842X.1999.tb01280.x 10462862

[B34] Miles MB Huberman A Qualitative Data Analysis: An Expanded Sourcebook 1994 Sage, London

[B35] Shin KR Kim MY Chung SE Methods and Strategies Utilized in Published Qualitative Research Qualitative Health Research 2009 19 6 850 858 10.1177/1049732309335857 19429769

[B36] Welsh E Dealing with Data: Using N-Vivo in the Qualitative Data Analysis Process Forum: Qualitative Social Research 2002 3 2

[B37] McCray T An issue of culture: the effect of daily activities on prenatal care utilisation patterns in rural South Africa Social Science and Medicine 2004 59 9 1843 1855 10.1016/j.socscimed.2004.02.033 15312919

[B38] Li J Gender inequality, family planning and maternal and child care in a rural Chinese county Social Science & Medicine 2004 59 4 695 708 10.1016/j.socscimed.2003.11.041 15177828

[B39] Matsuyama A Moji K Perception of Bleeding as a Danger Sign During Pregnancy, Delivery, and the Postpartum Period in Rural Nepal Qualitative Health Research 2008 18 2 196 208 10.1177/1049732307312390 18216339

[B40] Matthews Z Mahendra S Kilaru A Ganapathy S Antenatal Care, Care-seeking and Morbidity in Rural Karnataka, India: Results of a Prospective Study Asia-Pacific Population Journal 2001 16 2 11 28

[B41] Mumtaz Z Salway S I never go anywhere: extricating the links between women's mobility and uptake of reproductive health services in Pakistan Social Science & Medicine 2005 60 8 1751 1765 10.1016/j.socscimed.2004.08.019 15686807

[B42] Miles-Doan R Brewster KL The Impact of Type of Employment on Women's Use of Prenatal-Care Services and Family Planning in Urban Cebu, the Philippines Studies in Family Planning 1998 29 1 69 78 10.2307/172182 9561670

[B43] Sibley LM Sipe TA Koblinsky M Does traditional birth attendant training increase use of antenatal care? A review of the evidence Journal of Midwifery & Women's Health 2004 49 4 298 305 10.1016/j.jmwh.2004.03.009 15236709

[B44] Shakya K McMurray C Neonatal Mortality and Maternal Health Care in Nepal: searching for patterns of association Journal of Biosocial Science 2001 33 87 105 10.1017/S0021932001000876 11316397

[B45] Mullany BC Barriers to and attitudes towards promoting husbands' involvement in maternal health in Katmandu, Nepal Social Science and Medicine 2006 62 11 2798 2809 10.1016/j.socscimed.2005.11.013 16376007

[B46] DIFID HMGN Pregnancy and childbirth in Nepal 2004 http://www.nsmp.org/pregnancy_childbirth_nepal/index.html [online] Accessed on 18-11-09.

[B47] Karra MV Stark NN Wolf J Male involvement in family planning: A case study spanning five generations of a South Indian Family Study Family Planning 1997 28 1 24 34 10.2307/2137968 9097383

[B48] Dhakal S Chapman GN Simkhada PP van Teijlingen E Stephens J Raja AE Utilisation of postnatal care among rural women in Nepal BMC Pregnancy Childbirth 2007 7 19 17767710 10.1186/1471-2393-7-19 PMC2075509

[B49] Waszak C Thapa S Davey J The influence of gender norms on the reproductive health of adolescents in Nepal, perspective of youth Towards Adulthood, Exploring the Sexual and Reproductive Health of Adolescents in South Asia 2003 World Health Organization, Geneva

[B50] Allendorf K Couples' Reports of Women's Autonomy and Health-care Use in Nepal Studies in Family Planning 2007 38 1 35 46 10.1111/j.1728-4465.2007.00114.x 17385381

[B51] Furuta M Salway S Women's position within the household as a determinant of maternal health care use in Nepal International Family Planning Perspectives 2006 32 1 17 27 10.1363/3201706 16723298

[B52] Vlassoff C Gender inequalities in health in the Third World: uncharted ground Social Science and Medicine 1994 39 9 1249 59 10.1016/0277-9536(94)90357-3 7801162

[B53] Giddens A Bryant GA, Jary D Structuration theory: past, present and future Anthony Giddens' Theory of Structuration: A critical Appreciation 1991 Routledge, London 81 97

[B54] Kabeer N Resources, agency, achievements: reflections on the measurement of women's empowerment Development and Change 1999 30 3 435 464 10.1111/1467-7660.00125

[B55] Onega LL Stanhope M, Lancaster J Education theories, models and principles applied to community and public health nursing Community and public health nursing 2000 St. Louis: MO Mosby 266 283

[B56] Stephenson R Tsui AO Contextual influences on reproductive health service use in Uttar Pradesh, India Studies in Family Planning 2002 33 4 309 320 10.1111/j.1728-4465.2002.00309.x 12561780

[B57] Birbili M Translating from one language to another Social Research Update 2000 3 http://sru.soc.surrey.ac.uk/SRU31.html Accessed on 19-03-10.

[B58] Shrestha GM Lamichhane SR Thapa BK Chitrakar R Useem M Comings JP Determinants of Educational Participation in Rural Nepal Comparative Education Review 1986 30 4 508 522 10.1086/446633

[B59] Kerr RB Dakishoni L Shumba L Msachi R Chirwa M We Grandmothers Know Plenty: Breastfeeding, complementary feeding and the multifaceted role of grandmothers in Malaw Social Science & Medicine 2008 66 1095 1105 10.1016/j.socscimed.2007.11.019 18155334

